# Vancomycin-Induced DRESS Syndrome: An Important Concern in Orthopedic Surgery

**DOI:** 10.1155/2018/1439073

**Published:** 2018-06-24

**Authors:** Emma Littlehales, Odhrán Murray, Robert Dunsmuir

**Affiliations:** ^1^Department of Orthopedic and Spinal Surgery, Leeds General Infirmary, Leeds, UK; ^2^Department of Orthopedic and Spinal Surgery, Queen Elizabeth University Hospital, Glasgow, UK

## Abstract

DRESS (drug reaction with eosinophilia and systemic symptoms) is a potentially serious complication when prolonged courses of antibiotics are given to patients, with an average onset of 2–6 weeks after commencement. There is a high mortality rate (1–10%). We report the case of a 62-year-old male who developed DRESS after seven weeks of antibiotic treatment with vancomycin for a deep spinal metalwork infection. We describe the typical rash and biochemical results, including eosinophilia, as well as the systemic signs seen in this case. The criteria for diagnosis of DRESS, including the RegiSCAR scoring system and commonly affected systems (renal, cardiac, and hepatic), are detailed, and we also discuss evidence for steroid treatment and considerations important in the use of this.

## 1. Introduction

Drug reaction with eosinophilia and systemic symptoms (DRESS) syndrome was first named in 1996 by Bocquet et al. [1] and is known by a variety of different acronyms to describe a similar clinical picture [2]. There is a well-known association between the DRESS syndrome and many antiepileptic medications, but its association with vancomycin is less well documented. DRESS syndrome is a rare reaction to drugs, estimated to have an incidence between 1 in 1000 and 1 in 10,000 exposures, characterised by a severe skin reaction, fever, eosinophilia, or other haematological abnormalities and other organ involvement, most commonly the liver [3]. It usually occurs with a delayed onset from initiation of the causative drug of 2–6 weeks [1, 4] and undergoes a prolonged clinical course. It has a high mortality rate of 10% due to visceral organ involvement [3], although recent reports have put this as low as 1-2% [5].

## 2. Case

**Figure 1:**
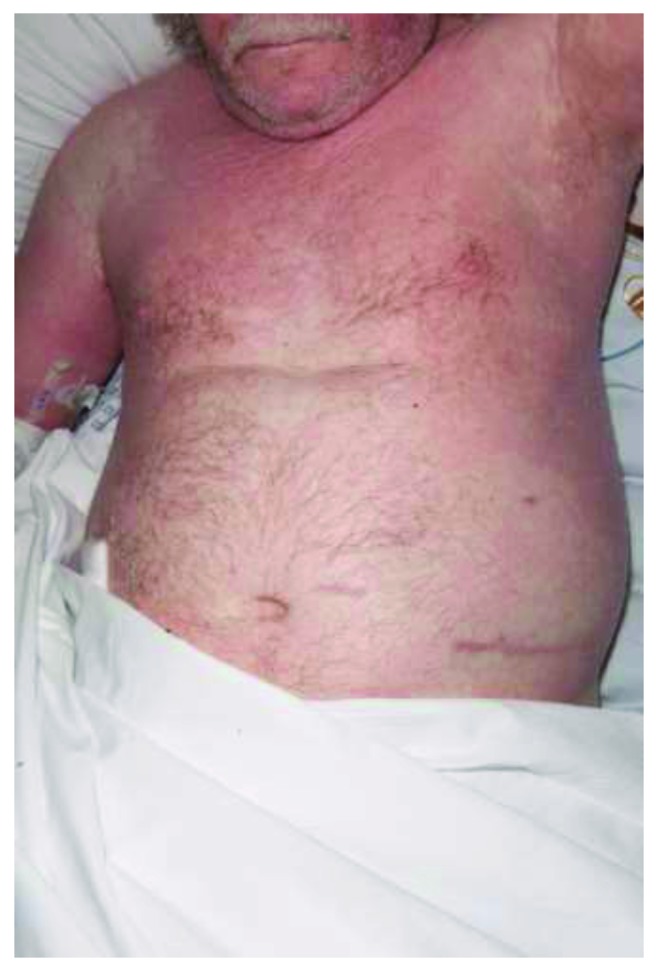
Initial distribution of the characteristic morbilliform rash of DRESS syndrome, progressing downwards from the face and arms. Also visible, left nephrectomy scar.

**Figure 2:**
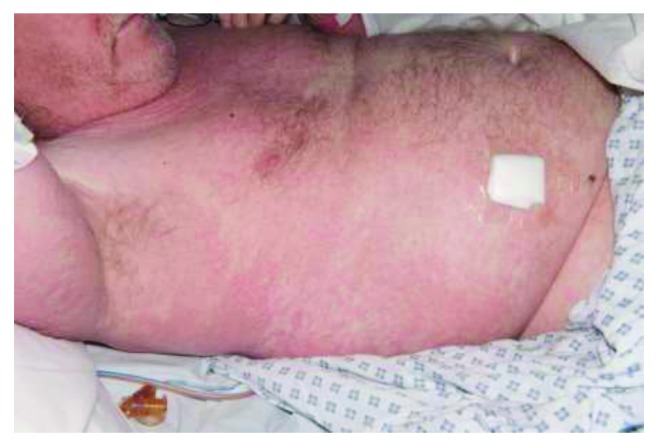
Progression of the macular rash down the body in a typical DRESS distribution.

**Figure 3:**
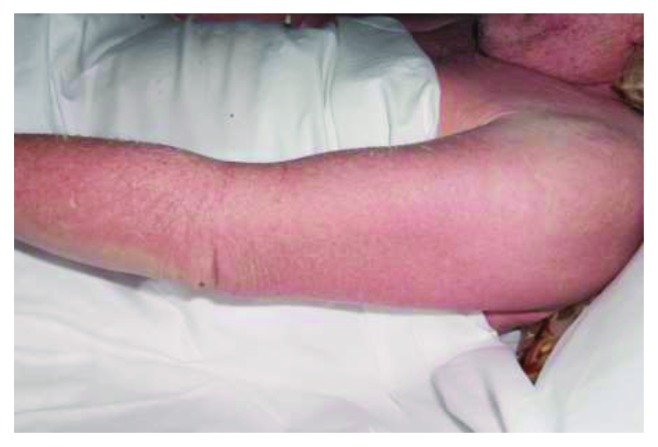
Progression of the rash to a confluent, exfoliative dermatitis after several days.

We report a case of a 62-year-old male who developed DRESS syndrome after seven weeks of antibiotic treatment with vancomycin. He initially underwent instrumented thoracic spinal fusion (T1–7) due to cord compression from a metastatic T4 lesion from renal cell carcinoma and developed a postoperative deep spinal infection. He underwent multiple washouts and vacuum-assisted closure over a period of twelve weeks, with various antimicrobial regimes, initially receiving seven weeks of vancomycin as well as a shorter duration of ciprofloxacin. He developed a maculopapular morbilliform rash, (Figure 1) initially on the right arm and scalp, before spreading to cover the entire head, trunk, and upper legs (Figure 2) which progressed to become exfoliative and was intensely pruritic and painful (Figure 3). This was accompanied by a fever and eosinophil count of 9.77 × 10^−9^/L at the highest, occurring simultaneously with the development of the rash, and which remained elevated over the course of a month of regular blood tests. Other haematological abnormalities were also present, with a rise in both lymphocytes and neutrophils. Vancomycin was discontinued immediately, and other causes for these results were excluded, with negative blood cultures, CMV, EBV, ANA, and hepatitis B, hepatitis C, and HIV titres. There was no clinically apparent lymphadenopathy; however, a CT scan performed after the onset of symptoms showed new prominent right hilar lymph nodes, although this may have been due to metastatic cancer and not DRESS syndrome. Skin biopsy showed superficial perivascular lymphocytic infiltrate and rare eosinophils, consistent with a morbilliform drug rash. Ciprofloxacin was felt to be unlikely to be the cause of his DRESS, as he had been prescribed the drug several times in the past, as well as having a shorter duration of treatment which would not fit with the typical timeframe for DRESS, so this was continued to treat his infection.

**Figure 4:**
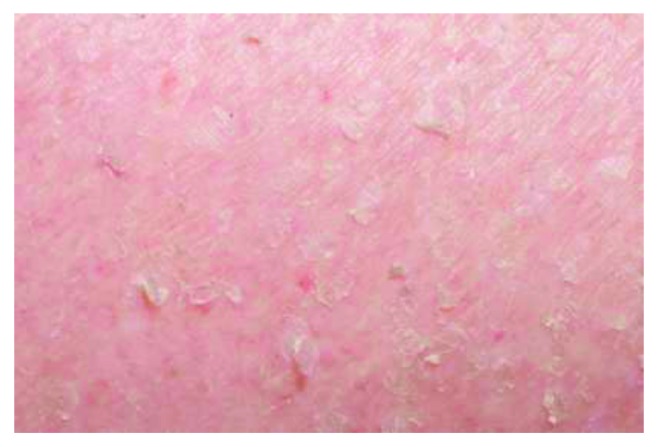
Exfoliative rash following treatment with topical steroids.

The patient initially received a single dose of intravenous high-dose hydrocortisone, but due to the severity of infection and the risk of immunosuppression, he was subsequently treated exclusively with topical steroids, emollients, and antihistamines (Figure 4). No liver or renal function abnormalities were noted during this time; however his eosinophils remained raised as described. He developed acute chest pain and shortness of breath four weeks after the initial rash, with new onset fast atrial fibrillation and negative troponin and creatinine kinase. A CT scan demonstrated bilateral pleural effusions, as well as progression of lung and rib metastases. An echocardiogram showed mild left ventricular and right ventricular impairment and a rim of pericardial fluid. Unfortunately, within three months of initial surgery, the metastatic spinal load increased causing further cord injury and paraplegia. Further surgical intervention was deemed inappropriate at this point, and the patient was discharged to the community palliative care team.

## 3. Discussion

DRESS syndrome is of particular concern in spinal surgery given the long courses of antibiotics that are given to patients with osteomyelitis and discitis and those with metalwork infections. Young et al. [6] reported on three cases of definite DRESS occurring with vancomycin in an orthopedic population in 2014, all after long-term treatment. Cacoub et al.'s literature review [3] found 2% of DRESS cases occurred with vancomycin, as well as isolated cases with other antibiotics including co-amoxiclav and streptomycin. In Kardaun et al.'s large multicentre series [5], 23% of cases were due to antibiotics. There are obviously difficulties in diagnosing DRESS, given the varied presentation and causative drugs, as well as there being no validated biochemical test for the syndrome [2]. The RegiSCAR scoring system [7] aims to standardise the DRESS diagnosis amongst healthcare providers (Table 1).

Our patient scored between 5 and 7 (probable/definite case). It is difficult to accurately differentiate if DRESS syndrome was the cause of the enlarged lymph nodes or cardiac symptoms seen, as these were not investigated, given the palliative nature of the patient's case.

The cause of DRESS is not fully understood. It is generally regarded, however, as a severe hypersensitivity reaction to a drug or its metabolites [4]. Most evidence points to an immune-mediated cause, with the reaction occurring after sensitisation and a more rapid onset after subsequent drug administration [4]. It has been shown to be related to immunosuppression, both as a risk factor and a result of the syndrome [4, 8], and may also be related to specific genetic mutations leading to enzymatic defects in drug metabolism pathways [2]. There is some evidence to suggest that human herpesvirus 6 is associated with DRESS syndrome [8]; however, it is unclear if this is a causative factor in DRESS or due to reactivation of the virus by autoimmune pathways and drug metabolites [4].

DRESS often initially presents with a pyrexia and pruritus, before development of a rash, starting on the face, upper trunk, and limbs before progressing down the body (Figures 1 and 2). It usually starts as a macular, morbilliform rash, present in 81%–97% [3, 9] and progresses to involve the entire body surface in an exfoliative dermatitis [4] (Figure 3). As would be expected given the nomenclature, haematological abnormalities are common, and leukocytosis is normally seen, although eosinophilia > 2.0 × 10^−9^/L is found in anywhere between 30% and 95% of cases [4, 5].

The high mortality rate is normally due to visceral organ involvement, almost exclusively the liver, although more rarely due to cardiac effects [3]. Other commonly affected systems include the kidneys and lungs, with rare GI, endocrine, and neurological involvement [4]. The liver is the most frequently involved organ in DRESS, seen in 94% of cases with internal organ involvement [3]. This usually takes the form of elevated alanine aminotransferase and can progress to severe acute hepatitis and hepatic necrosis leading to liver failure [4, 10].

Cardiac involvement was only seen in 2% of cases in Cacoub et al.'s review [3]. It is, however, poorly recognised and can occur with a delay of up to 4 months [10]. Acute eosinophilic myocarditis is the most common form, and this can progress to acute necrotising eosinophilic myocarditis. It is imperative that this second type is recognised early, as it has a mortality of >50%, with a median survival of 3-4 days [10]. Cardiac involvement normally presents with chest pain, tachycardia, and shortness of breath [4]. The first type tends to be self-limiting and is characterised by nonspecific ECG changes, sinus tachycardia, arrhythmias, systolic dysfunction, pleural effusions, and occasional pericardial effusion on echo [4, 10]. There is usually an effect on both ventricles. The definitive diagnosis of DRESS-induced myocarditis is an endocardial biopsy [10], which was not indicated in our patient due to the palliative nature of his cancer care. However, given the clinical, biochemical, and imaging results, it is likely that there was an element of cardiac involvement secondary to DRESS syndrome.

Renal involvement is generally asymptomatic, occurring in 8–11% [3, 4] of patients with DRESS syndrome and is identified by biochemical markers demonstrating impaired renal function. Renal impairment is also usually self-limiting but in severe cases can progress to severe interstitial nephritis. The long-term sequelae of DRESS syndrome include autoimmune conditions such as Grave's disease and type 1 diabetes, which may occur months to years following exposure to the causative drug [4].

Hussain et al. [11] recommend a broad screening for potential visceral effects of DRESS syndrome (Table 2).

The mainstay of treatment of DRESS syndrome is immediate withdrawal of the culprit drug, followed by systemic steroids [3, 11]; our patient received a single high dose of intravenous hydrocortisone along with rapid cessation of vancomycin. Topical steroids are considered the accepted treatment for cutaneous symptoms; however, there is limited evidence of benefit [12]. There is no level I–III trial evidence examining the route, type, or duration of systemic steroid treatment in DRESS syndrome, with a wide variety of doses reported in the literature [13]. Given the high risks, especially in a patient who has developed DRESS due to antibiotic treatment of a severe infection, it is difficult to formulate an accurate risk : benefit analysis. There is some data to suggest that patients suffering from DRESS syndrome may require months of systemic steroids [14] with the associated risks, including immunosuppression. It is notable that there is an absence of level I–III evidence into treatment modalities, and therefore, there is little consensus as to the most effective treatment.

The use of long-term vancomycin in those spinal surgery patients who develop infection is not uncommon, due to its spectrum of activity and bone penetration. The treating physician should have a high index of suspicion for DRESS syndrome for any patient within this cohort who develops a rash after several weeks of treatment. In the absence of high-level evidence, the risks and benefits of long-term systemic steroid treatment must be carefully considered, especially in the presence of indwelling metalwork. Clinical decision-making involves weighing up the deleterious systemic autoimmune effects of DRESS syndrome with the immunosuppressive effects of steroids in a condition with a long course and high mortality.

## Figures and Tables

**Table 1:** RegiSCAR scoring system for classifying cases of DRESS [7].

Score	−1	0	1	2
Fever > 38.4	No	*Yes*		
Enlarged lymph nodes		No	Yes	
Eosinophilia		No	0.7–1.499 × 10^−9^/L	*>1.5 × 10^−9^/L*
Atypical lymphocytes		No	Yes	
Skin rash > 50% body surface area		No	*Yes*	
Skin rash suggesting DRESS	No		*Yes*	
Biopsy suggesting DRESS	No	*Yes*		
Organ involvement		No	1 organ	2 or more organs
Resolution > 14 days	No	*Yes*		
Evaluation of other potential causes (>2 negative of ANA, blood culture, hepatitis A/B/C, and chlamydia/mycoplasma)			*Yes*	

Score: <2 no case, 2-3 possible case, 4-5 probable case, >5 definite case. The diagnostic criteria met by this case are highlighted in italics, totaling a score of 5. If the enlarged lymph nodes and cardiac involvement are taken to be as a result of DRESS, the score rises to 7.

**Table 2:** Algorithm for the diagnosis of visceral complications in DRESS syndrome [11].

Hepatic	LFTs PT/PTT/INR Hepatitis screening
Cardiac	ECG Echocardiogram Cardiac enzymes
Pulmonary	Chest X-ray Pulmonary function tests
Renal	U + Es, nitrogen Urinalysis Renal USS
Endocrine	TST/T4 Fasting glucose
Gastrointestinal	Fecal occult blood Lipase
Neurological	Head CT/MRI EEG CSF analysis

## References

[1] Bocquet H., Bagot M., Roujeau J. C. (1996). Drug-induced pseudolymphoma and drug hypersensitivity syndrome (drug rash with eosinophilia and systemic symptoms: DRESS). *Seminars in Cutaneous Medicine and Surgery*.

[2] Choudhary S., McLeod M., Torchia D., Romanelli P. (2013). Drug reaction with eosinophilia and systemic symptoms (DRESS) syndrome. *The Journal of Clinical and Aesthetic Dermatology*.

[3] Cacoub P., Musette P., Descamps V. (2011). The DRESS syndrome: a literature review. *The American Journal of Medicine*.

[4] Husain Z., Reddy B. Y., Schwartz R. A. (2013). DRESS syndrome. *Journal of the American Academy of Dermatology*.

[5] Kardaun S. H., Sekula P., Valeyrie-Allanore L. (2013). Drug reaction with eosinophilia and systemic symptoms (DRESS): an original multisystem adverse drug reaction. Results from the prospective RegiSCAR study. *The British Journal of Dermatology*.

[6] Young S., Ojaimi S., Dunckley H. (2014). Vancomycin-associated drug reaction with eosinophilia and systemic symptoms syndrome. *Internal Medicine Journal*.

[7] Kardaun S. H., Sidoroff A., Valeyrie-Allanore L. (2007). Variability in the clinical pattern of cutaneous side-effects of drugs with systemic symptoms: does a DRESS syndrome really exist?. *The British Journal of Dermatology*.

[8] Suzuki Y., Inagi R., Aono T., Yamanishi K., Shiohara T. (1998). Human herpesvirus 6 infection as a risk factor for the development of severe drug-induced hypersensitivity syndrome. *Archives of Dermatology*.

[9] Ang C.-C., Wang Y.-S., Yoosuff E.-L. M., Tay Y.-K. (2010). Retrospective analysis of drug-induced hypersensitivity syndrome: a study of 27 patients. *Journal of the American Academy of Dermatology*.

[10] Bourgeois G. P., Cafardi J. A., Groysman V., Hughey L. C. (2012). A review of DRESS-associated myocarditis. *Journal of the American Academy of Dermatology*.

[11] Husain Z., Reddy B. Y., Schwartz R. A. (2013). DRESS syndrome: part II. Management and therapeutics. *Journal of the American Academy of Dermatology*.

[12] Ghislain P. D., Roujeau J. C. (2002). Treatment of severe drug reactions: Stevens-Johnson syndrome, toxic epidermal necrolysis and hypersensitivity syndrome. *Dermatology Online Journal*.

[13] Jeung Y.-J., Lee J.-Y., Oh M.-J., Choi D.-C., Lee B.-J. (2010). Comparison of the causes and clinical features of drug rash with eosinophilia and systemic symptoms and Stevens-Johnson syndrome. *Allergy, Asthma and Immunology Research*.

[14] Walsh S. A., Creamer D. (2011). Drug reaction with eosinophilia and systemic symptoms (DRESS): a clinical update and review of current thinking. *Clinical and Experimental Dermatology*.

